# Immunotherapy, Inflammation and Colorectal Cancer

**DOI:** 10.3390/cells9030618

**Published:** 2020-03-04

**Authors:** Charles Robert Lichtenstern, Rachael Katie Ngu, Shabnam Shalapour, Michael Karin

**Affiliations:** 1 Department of Pharmacology, School of Medicine, University of California, San Diego, La Jolla, CA 92093, USAkarinoffice@health.ucsd.edu (M.K.); 2 Laboratory of Gene Regulation and Signal Transduction, Department of Pharmacology, School of Medicine, University of California, San Diego, La Jolla, CA 92093, USA; 3 Moores Cancer Center, University of California, San Diego, La Jolla, CA 92093, USA

**Keywords:** colorectal cancer, immunotherapy, inflammation, microsatellite instability

## Abstract

Colorectal cancer (CRC) is the third most common cancer type, and third highest in mortality rates among cancer-related deaths in the United States. Originating from intestinal epithelial cells in the colon and rectum, that are impacted by numerous factors including genetics, environment and chronic, lingering inflammation, CRC can be a problematic malignancy to treat when detected at advanced stages. Chemotherapeutic agents serve as the historical first line of defense in the treatment of metastatic CRC. In recent years, however, combinational treatment with targeted therapies, such as vascular endothelial growth factor, or epidermal growth factor receptor inhibitors, has proven to be quite effective in patients with specific CRC subtypes. While scientific and clinical advances have uncovered promising new treatment options, the five-year survival rate for metastatic CRC is still low at about 14%. Current research into the efficacy of immunotherapy, particularly immune checkpoint inhibitor therapy (ICI) in mismatch repair deficient and microsatellite instability high (dMMR–MSI-H) CRC tumors have shown promising results, but its use in other CRC subtypes has been either unsuccessful, or not extensively explored. This Review will focus on the current status of immunotherapies, including ICI, vaccination and adoptive T cell therapy (ATC) in the treatment of CRC and its potential use, not only in dMMR–MSI-H CRC, but also in mismatch repair proficient and microsatellite instability low (pMMR-MSI-L).

## 1. Introduction

Colorectal cancer (CRC) is the third most common cancer type and a leading cause of mortality among cancer-related deaths in the United States [1]. While scientific and clinical advances in early detection and surgery have led to five-year survival rates of 90% and 71% for localized and regionalized CRCs, respectively, the five-year survival rate for metastatic CRC is low, remaining at around 14% [2]. Moreover, 25% of CRC patients display metastasis at diagnosis, and roughly 50% of those treated will eventually develop metastasis during their lifetime [3]. These alarming statistics can most likely be attributed to the ineffectiveness of standard treatment regimens, and thus indicates an urgent need for the development of more effective treatment options. Immunotherapy, a treatment option that takes advantage of the body’s own immune system to attack cancer, has shown promise in the treatment of certain cancers [4,5,6,7]. Whereas some cancers, such as melanoma and lung cancer, respond well to immune checkpoint inhibitor therapy (ICI), others do not.

More recently, ICIs were found effective in a specific subset of CRC that is mismatch-repair-deficient (dMMR) and microsatellite instability-high (MSI-H) (referred to as dMMR-MSI-H tumors) and ineffective in subsets that are mismatch-repair-proficient (pMMR) and microsatellite instability-low (MSI-L) (referred to as pMMR-MSI-L tumors) [8]. This Review will serve to discuss recent findings in the effectiveness of immunotherapies in the treatment of CRC, both localized and metastatic, from clinical trials and experimental models, and its potential use in pMMR-MSI-L tumors and other CRC subsets.

## 2. Origins of CRC

**Figure 1:**
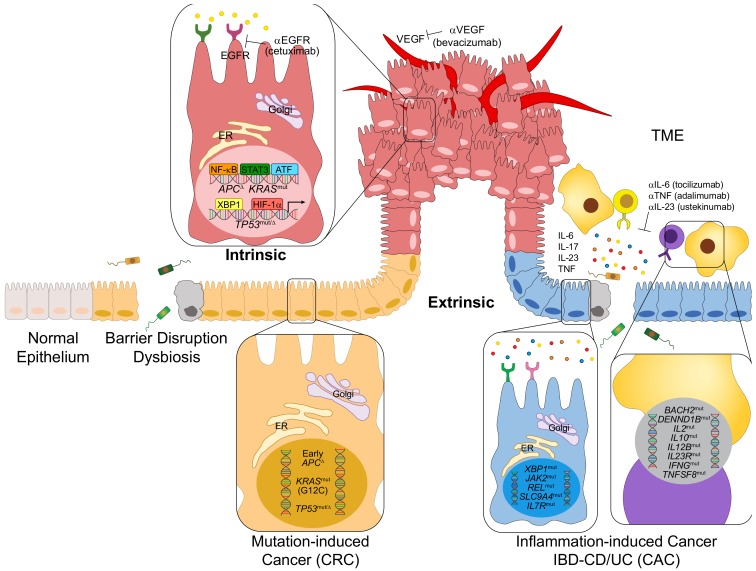
Intrinsic and extrinsic factors contributing to the pathogenesis of colorectal cancer (CRC). CRC can develop from a multitude of both intrinsic and extrinsic factors. Extrinsic factors, including inflammation from hyperactivated immune cells, the release of proinflammatory cytokines, or gut dysbiosis, can lead to an inflammatory and possibly premalignant environment. Intrinsic factors include sporadic mutations, such as those leading to mutation-induced CRC (sporadic CRC). Similarly, precancerous mutations, or mutations induced by prior inflammation, can lead to colitis-associated cancer (CAC), a specific subset of CRC stemming from chronic inflammation caused by inflammatory bowel disease (IBD), specifically ulcerative colitis (UC) or Crohn’s disease (CD).

CRC can originate from a multitude of intrinsic and extrinsic factors, including an accumulation of new mutations, pre-existing mutations, and susceptibility alleles associated with family history, or chronic, lingering inflammation, as described in Figure 1. The majority (75%) of CRCs are sporadic, meaning family history is not involved in their pathogenesis [9]. Common mutations in tumor suppressor genes and oncogenes that give rise to CRC include adenomatous polyposis coli (*APC*), tumor protein 53 (*TP53*), and Kirsten rat sarcoma (*KRAS*), which are present in 81%, 60% and 43% of the cases of sporadic CRCs, respectively [10]. The role of these genetic alterations in the pathogenesis of CRC has been extensively reviewed [11,12,13]. Most CRC-inducing mutations act in a particular order, controlling the adenoma–carcinoma sequence, which describes the progression of a normal intestinal epithelia to an adenoma, invasive carcinoma, and eventual metastatic tumor [14,15].

Family history is implicated in approximately 10–30% of CRCs [16,17]. For example, familial adenomatous polyposis (FAP) and hereditary nonpolyposis colorectal cancer (Lynch syndrome) are the most commonly inherited CRC syndromes, and account for 2–4% and 1% of CRC cases, respectively [17].

Although 96% of all CRCs do not develop in the context of pre-existing inflammation, the roles of chronic inflammation, tumor-elicited inflammation, the tumor microenvironment (TME), and partially adaptive immune cells in CRC development, have been established, particularly in the context of their interaction with gut dysbiosis [18,19,20,21,22,23]. Colitis-associated cancer (CAC) is a specific subset of CRC characterized by its implication with inflammation that accounts for 1%–2% of all CRCs [24]. CAC, originating from either the chronic inflammation in both the colon and the small intestine, or solely the colon, as is the case of Crohn’s disease (CD) or ulcerative colitis (UC), respectively, is classified by the excessive activation and recruitment of immune cells that produce inflammatory cytokines, such as TNF, IL-17, IL-23 and IL-6, that lead to the propagation of an inflammatory and possibly premalignant environment [25]. Mutations involved in inflammatory bowel disease (IBD) development include genes that regulate immune activation and the subsequent response, such as *IL12B*, *IL2*, *IFNG*, *IL10*, *TNFSF8*, *TNFSF15*, *IL7R*, *DENND1B*, *JAK2* and those that also regulate ER stress, glucose, bile salt transfer and organic ion transporter, including *XBP1*, *SLC9A4*, *SLC22A5* and *SCL11A1*, as shown in Figure 1 [26]. Both CRC and CAC exhibit inflammatory microenvironments, but the order in which inflammation and tumorigenesis occur seems to be different. In CRC, inflammation follows tumorigenesis. Mutations due to environmental factors initiate tumor development in CRCs, and the subsequent activation of inflammatory cells can induce further DNA damage through the production of reactive oxygen species (ROS) and reactive nitrogen intermediates (RNIs) [25,27]. On the other hand, inflammation precedes tumorigenesis in CAC. Inflammation induced by the activation of immune cells and their release of proinflammatory cytokines can induce DNA damage and mutations in CAC [25]. Correspondingly, both CRC and CAC may entail similar mutations, but the timing and order of these mutations are different, as displayed by early *APC* and late *TP53* mutations in CRC, and early *TP53* and late *APC* mutations in CAC [28,29,30]. Another important contributor to CRC emergence is so-called tumor-elicited inflammation driven by the loss of normal barrier function as a result of *APC* inactivation [18].

## 3. Mismatch Repair Deficiency and Microsatellite Instability in CRC

dMMR or MSI-H exists in about 15% of all cases of CRC, but only in 4% of metastatic CRC, as opposed to pMMR or MSI-L, which is present in roughly 85% of all cases of CRC. MSI occurs in both spontaneous CRC and IBD-induced CAC, although the rates and timing at which MSI occurs are similar in both malignancies [31].

**Figure 2:**
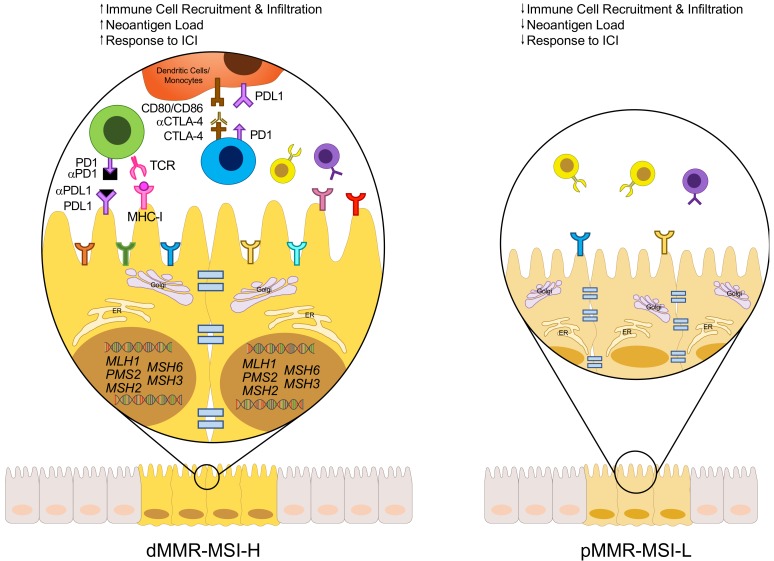
Immuno-landscape of dMMR-MSI-H and pMMR-MSI-L CRC. CRC can be classified into two subsets based on its MMR/MSI status. The DNA MMR system relies on key genes, such as MLH1, MSH2, MSH6, PMS2, or MSH3, that correct mismatched or wrongly inserted or deleted bases in the DNA. If this machinery fails due to defects in one or more of the repair genes, these errors are free to be integrated into the DNA permanently, forming microsatellites. Thus, dMMR-MSI-H tumors are those that have a defect in one of the major DNA repair genes (dMMR), resulting in high levels of microsatellites (MSI-H). On the other hand, pMMR-MSI-L tumors have a functional MMR system (pMMR), resulting in low or stable levels of microsatellites (MSI-L). The result of this damaged repair system in dMMR-MSI-H tumors is a higher mutational burden, which correlates with a higher expression of neoantigens on MHC-I molecules.

Microsatellites are repetitive DNA sequences that can experience a sudden and prolonged change in size, due to errors during DNA replication, such as the formation of small loops in the DNA strands, leading to MSI-H [32]. These errors are combated by the mismatch repair (MMR) system, an ancient mechanism used to correct insertions, deletions, or mismatched bases that are generated by the erroneous loops that form during DNA replication [32,33,34]. However, if there is a dysfunction or mutation in the MMR system, referred to as dMMR, these errors are left uncorrected, allowing them to be integrated into the DNA permanently [32]. Thus, MSI-H tumors have varied lengths of microsatellites (compared to MSI-L) due to errors in the MMR system, as shown in Figure 2.

The MMR system relies on the DNA repair genes *MLH1*, *MSH2*, *PMS1*, *MSH6*, *PMS2* and *MSH3*, all of which are involved in correcting mismatched or wrongly inserted or deleted bases in DNA [32,35]. Loss, inactivation, or the silencing of any one of these genes, classifies a patient as dMMR. More importantly, errors in this repair system lead to a high mutational profile, which explains why dMMR tumors have an average mutational profile of 1782, compared to 73 for pMMR tumors [36].

As the identification and classification of CRCs is necessary and crucial for proper diagnoses and treatments, methods have been practiced in order to detect MSI. Current methods include the amplification and examination of polymerase chain reaction (PCR) products from commonly affected microsatellite markers in tumors [34,37,38]. These markers include two mononucleotide repeat markers (BAT-25 and BAT-26) and three dinucleotide repeat markers (D2S123, D5S346, and D17S250) [37]. MSI-H status is classified if instability is present in two or more of the markers, whereas the MSI-L status is classified if instability is only detected in one of the markers. More recently, however, are methods that use DNA-sequencing technology for MSI detection and classification on the same markers [33,39,40]. Regardless of the screening method, albeit some more efficient and accurate than others, classification of MSI status in regard to the CRC subtype is of the upmost importance for proper treatment planning, and should be one of the primary steps when diagnosing patients.

## 4. Classical Treatment Options

CRC treatment can be divided into two main treatment categories: neoadjuvant and adjuvant. Neoadjuvant therapy refers to therapeutics that are given before the main cancer treatment, usually surgery, whereas adjuvant refers to that which is given after or in combination with the main cancer treatment. Neoadjuvant therapy offers many clinical benefits, in that it can potentially lessen the severity of the malignancy, through eliminating early metastatic tumors, preventing complications during surgery, and allowing for a more accurate plan for adjuvant therapy (if necessary), based on the subsequent response to neoadjuvant therapy [41,42,43]. Most studies have shown that neoadjuvant chemotherapy may improve overall survival, depending on the severity and stage of the disease [41,44,45,46].

Chemotherapy is usually the first line of defense in the treatment of CRC. 5-fluorouracil (5-FU), the most common of the chemotherapeutic agents for CRC, acts through inhibition of thymidylate synthase, which converts deoxyuridine monophosphate (dUMP) to deoxythymidine monophosphate (dTMP), causing DNA damage [47]. While it is relatively effective in early disease stages, response rates in metastatic CRC are only 10–15% [47,48]. On the other hand, combinatorial chemotherapeutic regimens consisting of 5-FU, in combination with oxaliplatin (FOLFOX) or irinotecan (FOLFIRI), have heightened response rates to 40–50% [47]. Studies into the usefulness of using MMR/MSI status as a predictor of responsiveness to chemotherapy have shown mixed results, depending on the stage of the disease and the specific type of chemotherapy, thus explaining the necessity for a more reliable and dependable treatment option for these CRC subsets [49,50,51,52,53].

More recently introduced are the targeted therapies, including monoclonal antibodies against epidermal growth factor receptor (EGFR) and vascular endothelial growth factor (VEGF), which inhibit cancer cell proliferation and angiogenesis, respectively. Bevacizumab, a monoclonal antibody against VEGF, was shown to improve the survival of patients with metastatic CRC in combination with 5-FU [54] and oxaliplatin-based therapies [55]. Moreover, patients with irinotecan- [56] and fluoropyrimidine- and oxaliplatin-resistant [57] CRCs were shown to have improved response rates when treated with cetuximab, a monoclonal antibody against EGFR, alone or in combination with irinotecan. Extensive research has shown *KRAS* mutational status to be a predictor of non-responsiveness to EGFR inhibitors [58,59,60]. It was found that patients with pMMR tumors that had mutations in *BRAF* or *KRAS*, had worse survival rates than patients with pMMR tumors free of these mutations, and patients with dMMR tumors [61]. Despite major scientific and clinical research into targeted therapies, patients that do respond to EGFR inhibitors only show improvements for 3–12 months before disease progression, suggesting that this specific therapy is not conducive to long term survival and remission [56,58,62,63]. This obstacle has paved the way for research into the efficacy of immunotherapy in the treatment of CRCs.

## 5. Role of Immune Cells and Tumor Microenvironment in the Classification of CRC

A positive correlation is seen between tumoral CD3^+^ and CD8^+^ T cell densities and the risk of recurrence, disease-free survival rate, and the overall survival rate in patients with different stages of CRC [64]. This is in accordance and supports evidence which shows that increased amounts of tumor-infiltrating lymphocytes correlate with an improved clinical outcome and prognosis [65,66,67,68]. Both dMMR-MSI-H and pMMR-MSI-L tumors have distinctly different TME makeups and distributions of immune cell populations, contributing to the variation in response rates to therapy, treatment targets and clinical prognoses [69,70,71]. Comparison of the makeup of the TME shows a higher expression of cytotoxic, Th1, Th2, CD8^+^ T and follicular helper (Tfh) cell markers, in addition to macrophages and B cells in dMMR-MSI-H tumors than pMMR-MSI-L tumors [69,72]. Some of these immune cells can mediate antitumor immune responses, thus explaining why dMMR-MSI-H tumors have better response rates and clinical outcomes [73]. Higher mutational load in dMMR-MSI-H tumors correlates with the higher expression of neoantigens on major histocompatibility complex (MHC)-I molecules, thus recruiting more cytotoxic CD8^+^ T cells for the subsequent immune response and tumor destruction, which follows the notion that frameshift mutations positively correlate with CD8^+^ T cell infiltration in CRCs [74,75].

Since T cell infiltration is representative of a better clinical outcome in CRC patients, it is clear why dMMR-MSI-H tumors respond well to ICI, and pMMR-MSI-L tumors do not [76,77].

In addition to the wide variety of immune cells distributed throughout the TME, there are also many cytokines and other molecules secreted by these cells that have specific roles in inflammation, immunity and CRC development. These cytokines can have both antitumorigenic properties, such as interferon-gamma (IFN-γ) and granulysin, or pro-tumorigenic properties, such as IL-6, IL-23 and IL-17. IFN-γ [78] and granulysin [79] bolster and induce MHC-I antigen processing and presentation machinery, and they also recruit antigen presenting cells to stimulate tumor destruction, thus showcasing their antitumorigenic functions, and as so, are overexpressed in dMMR-MSI-H tumors [69]. Induction of proinflammatory cytokines originates as a result of NF-κB and STAT3 activation in epithelial cells, and serves an important role in supporting colorectal tumorigenesis [80,81,82]. IL-6 is overexpressed in CRC [83,84,85], and serves a pro-tumorigenic function through multiple processes, including bolstering angiogenesis through an enhanced expression of VEGF [86], protecting both healthy and malignant intestinal epithelial cells (IECs) from damage-associated molecular patterns (DAMPS), and pathogen-associated molecular patterns (PAMPS), by supporting their growth and survival [87,88,89,90,91], along with bolstering defects in the DNA MMR system [92]. Ablation of IL-6 in the dextran sodium sulfate/azoxymethane (DSS/AOM) mouse model of CRC resulted in diminished tumorigenesis, thus confirming its pro-tumorigenic properties [87]. Both IL-23 and IL-17 have also been implicated in the pathogenesis of CRC in human and murine models. An upregulation of IL-17 and IL-23 expression was found in tumors excised from the CPC-APC mouse model of CRC [18]. IL-23 enhances the production of IL-17, and IL-17 activates NF-κB which stimulates the proliferation and survival of IECs, resulting in accelerated colorectal tumorigenesis [18,19,80]. Correspondingly, the elevated expression of IL-6, IL-23 and IL-17 in CRC correlates with a worse prognosis and clinical outcome [93]. The role of other immunomodulatory cytokines involved in CRC has also been discussed [81,82,94]. The presence of a wide variety of immune cells and other cytokines and signaling molecules in the TME provide important topics for future research, but most importantly can serve as new possible targets for immunotherapy.

Moreover, CAC presents a different immuno-profile compared to CRC, which may increase the responsiveness to immunotherapy. However, it may also increase the risk for immunopathological side effects.

## 6. Why Immunotherapy?

Immunotherapy, particularly ICI, has revolutionized cancer treatment, and although response rates rarely exceed 20%, those who do respond show a durable response [95,96,97]. The responsiveness to ICI was suggested to depend on several key factors, including mutational load (high levels of tumor neoantigens), tumor-infiltrating lymphocytes and regulatory checkpoint receptors. ICI, a specific type of immunotherapy, functions through inhibiting negative regulatory receptors, such as cytotoxic T lymphocyte antigen 4 (CTLA4) and programmed cell death 1 (PD-1), on T cells, and thereby boosts antitumor immune responses [98,99,100,101]. T cells enable the immune system to recognize foreign antigens through an interaction between their T cell receptors (TCR) and peptide epitopes presented by MHC-I molecules on tumor cells [102,103]. Thus, it was suggested that cancers that are characterized by high mutational profiles can produce and present more neoantigens via their MHC-I molecules, and thereby lead to recognition, T cell activation and eventual self-destruction [8,104,105]. However, these effector T cells can become exhausted due to prolonged antigen stimulation, or through an interaction between their surface PD-1 with PD-L1 expressed by immune cells or tumor cells, or their surface CTLA-4 with CD80/CD86 expressed by dendritic cells, which are professional antigen-presenting cells (DC-APC) [101]. Inhibition of these interactions has been observed to partially reactivate exhausted T cells and induce tumor regression [106]. Higher response rates in non-small cell lung cancer (NSCLC) [104,107] and melanoma [108,109,110] have been attributed to the higher mutational loads in these tumor types [111].

However, for some tumors with lower inflammation and T cell infiltration, which could be due to defects on priming or the absence of high affinity T cells, vaccinations or more specific approaches like adoptive T cell therapy (ATC), which are specific for a particular mutated antigen, may prove favorable options in combination with ICI.

Therapeutic cancer vaccines can induce an immune response through a direct stimulation of the immune system by delivering antigens to DC-APC, which prime and activate CD4^+^ and CD8^+^ T cells to initiate tumor destruction [112]. Therapeutic cancer vaccines can target tumor-associated antigens (TAAs) or tumor-specific antigens (TSAs). TAAs are self-antigens expressed in both tumor and normal cells, however T cells that bind to these self-antigens can be removed from the immune system through immunotolerance mechanisms [113]. On the other hand, TSAs are unique to tumor cells, and can strongly induce an immune response through the binding and activation of T cells [113]. However, cancer vaccines comprising TSAs present a noticeable limitation, the necessity for a personalized vaccine specific to the individual’s particular tumor neoantigen.

Moreover, ATC provides tumor-antigen-specific approaches that have been shown to have promising results, and may be useful when the neoantigen load is lower, or if information regarding this neoantigen load is unavailable [114]. For example, CD8 T cells targeting mutant KRAS [115] or TP53 “Hotspot” Mutations [116] have been identified. Moreover, circulating PD-1^+^ lymphocytes have recently been shown to recognize human gastrointestinal cancer neoantigens [117].

## 7. Is There a Place for Immunotherapy in CRC?

Initially, ICI was not considered a viable treatment option for CRC. An initial phase II study assessed the efficacy of tremelimumab, a monoclonal antibody against CTLA4, in patients with previous treatment-refractory CRC, which resulted in no improvement post-treatment [118]. Furthermore, two phase I studies of anti-PD-1 [119] and anti-PD-L1 [120] antibodies in previously-treated CRC patients produced no responses. Unfortunately, the MMR/MSI status of the patients in both of these studies was unknown, compromising the interpretation of the results. Indeed, a subsequent phase I clinical trial of an anti-PD-1 antibody (MDX-1106) in patients with a variety of treatment-resistant tumors, including one patient with CRC, culminated in the patient achieving a durable complete response [121]. In accordance with the understanding that the response to ICI may correlate with mutational burden, Le et al. postulated that CRC tumors that are characterized by high mutational burdens due to mismatch–repair deficiencies may respond to ICI [36]. The results of the study showed that patients with dMMR-MSI-H tumors had a 40% objective response rate when treated with pembrolizumab, as compared to 0% for patients with pMMR-MSI-L tumors, and also exhibited 78% immune-related progression-free survival [36]. Importantly, these results suggested that the MMR/MSI status can be an accurate predictor of responsiveness to ICI using pembrolizumab.

Currently, a plethora of clinical trials aim to further examine ICIs in combination with a variety of other therapeutics in the treatment of CRC. Progress has led to United States Food and Drug Administration (FDA) approval of pembrolizumab and nivolumab in patients with dMMR-MSI-H CRC. Approval of pembrolizumab followed the results of the aforementioned study, being the first FDA approval based on a genetic biomarker of a particular tumor type [36]. Approval of nivolumab in patients with dMMR-MSI-H CRC followed the results of CheckMate-142, which showed a 31% objective response rate and 73% twelve month overall survival rate in treatment-resistant dMMR-MSI-H CRC [122]. This same trial also examined the efficacy of the combination of nivolumab and ipilimumab in treatment-resistant dMMR-MSI-H CRC, resulting in a 55% objective response rate and 85% twelve month overall survival rate [123]. The results of this study paved the way for FDA approval of that ICI combination in treatment-resistant dMMR-MSI-H CRC.

As the responsiveness to immunotherapy is generally associated with mutational load, as discussed previously, and dMMR-MSI-H patients comprise high mutational profiles, vaccinations targeting individuals’ unique neoantigens may prove to be effective, specifically in dMMR-MSI-H patients. In a murine model of induced dMMR by knockout of MLH1, vaccination extended overall survival and reduced the tumor burden, proving that vaccination can be a viable option for treatment in mouse models of dMMR [124].

Similarly, human clinical trials of therapeutic cancer vaccines have shown promising results depending on MSI status [125,126]. Ultimately, the main question to be determined is whether the combination of ICI and vaccination may prove to be more efficacious than ICI alone in dMMR-MSI-H CRC, or have the ability to elicit a response in pMMR-MSI-L CRC, which is unresponsive to ICI alone.

## 8. ICI-Resistance in pMMR-MSI-L CRC

Despite its effectiveness in dMMR-MSI-H CRC, ICI is not effective in pMMR-MSI-L CRC. The lack of response of pMMR-MSI-L tumors to ICI has been suggested to trace back to the diminished antitumor immune response, due to the inability for recognition by immune cells as a result of the low mutational profile of these tumors. This lack of response was also shown to be consistent in mouse models, as mice injected with MSI-H CRC experienced greater tumor regression and T cell infiltration than MSI-L or MSI-intermediate CRC when treated with anti-PD-1 therapy [127].

Although MSI-L tumors do not respond to ICI, higher T cell infiltration in MSI-L CRC is correlated with better disease free survival, indicating that some of these tumors can be recognized by T cells [64]. Thus, the main question to be discussed is whether MSI-L CRC utilizes other mechanisms to escape immunorecognition. Perhaps those patients with higher T cell infiltration can be selected for responsiveness to ICI.

One phase 3 trial examined the combination of cobimetinib, an MEK inhibitor, with atezolizumab, an anti-PD-L1 monoclonal antibody, in patients with metastatic CRC [128]. MEK inhibition resulted in increased amounts of tumor-infiltrating CD8^+^ T cells, and the combination with anti-PD-L1 treatment potentiated tumor regression in mouse models [129]. Despite the promising data in mouse models, the phase 3 trial failed to reach improved response or survival [128], leading to the conclusion that even when combined with MEK inhibitors, anti-PD-(L)1 is not effective in low immunoscore tumors, such as pMMR-MSI-L.

## 9. Conclusion: Thoughts, Obstacles, and Future Possibilities

**Figure 3:**
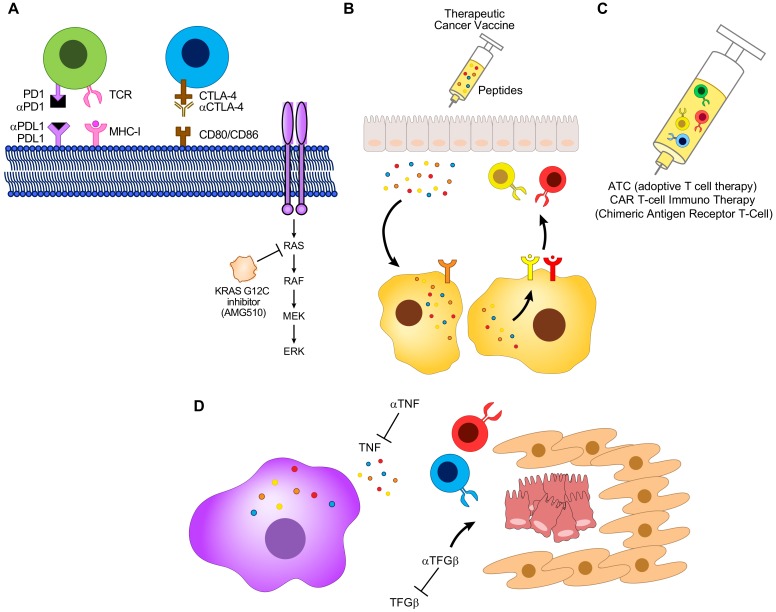
The future of CRC therapy: combinatorial agents. The current status of the use of inhibitor therapy (ICI) in the treatment of CRC has shown promising results, despite the lack of a complete response in dMMR-MSI-H tumors, and no response in pMMR-MSI-L. This obstacle has paved the way for insight and research into plausible combinatorial agents that can overcome this scientific impediment. (**A**) ICI in combination with AMG 510, a KRAS (G12C) inhibitor, or (**B**) therapeutic cancer vaccines, or (**C**) adoptive T cell therapy, or (**D**) TNF and TGFβ inhibitors may serve as the next candidates for combinatorial therapy with ICI.

CRC is a highly multifaceted and complex disease with an extensive mutational signature and an intricate TME. Just as complex as the disease itself, are the therapies used to combat it. Despite ICI’s initial effectiveness in patients exhibiting dMMR-MSI-H tumors, not all dMMR-MSI-H responds to ICI, and as of yet, no response is seen in pMMR-MSI-L. This has led to the necessity for new combinatorial targets that can be used to further bolster the response or lack of response to ICI in these two CRC subsets, as described in Figure 3. Recent advances in the development of new CRC therapeutics include AMG 510, a KRAS(G12C) inhibitor [130]. Analysis of AMG 510 in mouse models with *KRAS^G12C^*-injected tumors resulted in tumor regression, and combining this molecule with chemotherapy (carboplatin) or ICI (anti-PD-1) resulted in a further increase in tumor regression [130]. Analysis of these tumors showcased increased amounts of CD8^+^ T cells, macrophages and DC-APC in both the AMG 510 alone, and combination with anti-PD-1 treatment groups [130]. Clinical trials with AMG 510 in four patients with NSCLC resulted in objective partial responses and stable disease in two patients each [130]. The two partial responders were unresponsive to previous chemotherapy and ICI treatment, but exhibited tumor reduction of 34% and 67% when treated with AMG 510 [130]. Overall, this data suggests that AMG 510 may have the ability to induce T cell recruitment, and thus potentiate antitumor immunity. Whether AMG 510 can be combined with ICI in the treatment of CRC remains to be seen.

Cancer vaccines are a rapidly expanding immunotherapeutic approach that also seeks to exploit the body’s immune system to fight cancer. Therapeutic cancer vaccines can stimulate and activate T cells to initiate an immune response through the detection of TAAs or TSAs specific to the individuals’ tumors. Cancer vaccinations have shown mixed results in different stages of CRC, and more research is needed to truly uncover benefits [131,132,133]. Moreover, studies have shown that vaccinations may be efficacious in dMMR-MSI-H tumors, but not pMMR-MSI-L [126]. More interestingly, recent studies suggest that the combination of both ICI and cancer vaccinations may result in an improved response in some cancers, but not others [134,135,136,137,138,139,140,141,142,143,144]. Expanding on the possible immunotherapeutic options available, ATC may also provide a possible route in treating specific pMMR-MSI-L CRCs comprising KRAS mutations [145]. However, more research is warranted to determine its effectiveness in CRC, particularly dMMR-MSI-H and pMMR-MSI-L CRC in combination with ICIs. Just as CRC followed melanoma and NSCLC in its application in ICI therapy, CRC may be the next poster boy for vaccination and ICI combination-based therapy.

There are also a number of other factors that can modulate the response to ICI. The gut microbiome has been implicated in variations in response rates to ICI. The presence of particular microbiota seems to be correlated with a heightened response to ICI, depending on the strain and cancer type [146,147,148,149,150].

A possible adverse side effect common with ICI is immune-related colitis, which is often treated with antitumor necrosis factor α (TNF) antibodies. Such treatment in combination with ICI has been shown to improve antitumor immune responses and the severity of colitis in mouse models [151,152].

Furthermore, transforming growth factor β (TGFβ) signaling has been shown to cause resistance to ICI, and inhibition of TGFβ signaling in combination with ICI led to greater tumor regression, as opposed to ICI alone in mouse models, by inhibiting the cancer-associated fibroblast, and increasing the accessibility of cancer cells to T cells [153,154] (Figure 3D). These are just three further examples of possible factors that may be utilized to produce a better response to ICI in CRC.

Immunotherapy serves as a groundbreaking step towards new and more rational treatment options, and lay the groundwork for new combinatorial agents. Further research should be conducted to investigate new combinations of treatments that can be used to produce an improved response to ICI in dMMR-MSI-H CRC, and furthermore, a response that has not yet been obtained in pMMR-MSI-L CRC.
